# Efficacy and safety of guselkumab in patients with active lupus nephritis: results from a phase 2, randomized, placebo-controlled study

**DOI:** 10.1093/rheumatology/keae647

**Published:** 2024-12-02

**Authors:** Hans-Joachim Anders, Tak Mao Chan, Jorge Sanchez-Guerrero, David Wofsy, Karen Bensley, Lilianne Kim, Kim Hung Lo, Cathye Shu, Jie Shao, Chetan S Karyekar, Betty Diamond

**Affiliations:** Division of Nephrology, Department of Medicine IV, Hospital of the Ludwig-Maximilians University, Munich, Germany; Department of Medicine, School of Clinical Medicine at the University of Hong Kong, Hong Kong, China; Departmento de Inmunología y Reumatología, Instituto Nacional de Ciencias Medicas y Nutricion Salvador Zubiran, Mexico City, Mexico; Sinai Health System and University Health Network, Division of Rheumatology, University of Toronto, Toronto, ON, Canada; University of California San Francisco, San Francisco, CA, USA; Janssen Research & Development, LLC, Spring House, PA, USA; Janssen Research & Development, LLC, Spring House, PA, USA; Janssen Research & Development, LLC, Spring House, PA, USA; Janssen Research & Development, LLC, Spring House, PA, USA; Janssen Research & Development, LLC, Spring House, PA, USA; Janssen Research & Development, LLC, Spring House, PA, USA; The Center for Autoimmune, Musculoskeletal and Hematopoietic Diseases, The Feinstein Institutes for Medical Research, Manhasset, NY, USA

**Keywords:** lupus nephritis, biologics, guselkumab

## Abstract

**Objective:**

Evaluate the efficacy and safety of guselkumab, an IL-23p19-subunit inhibitor, in a phase 2, multicentre, randomized, double-blind, placebo-controlled study of patients with active LN.

**Methods:**

Adults (18–75 years) with active LN [Class III–IV proliferative nephritis (kidney biopsy) and urine protein-to-creatinine ratio (UPCR) of ≥1 mg/mg despite standard-of-care therapy] were randomized (1:1; planned sample = 60) to receive i.v. infusions of guselkumab 400 mg or placebo at weeks 0, 4 and 8, then s.c. injections (guselkumab 200 mg or placebo) at week 12 and every 4 weeks through week 48 in addition to their background therapy. The primary endpoint was achievement of ≥50% decrease in proteinuria from baseline at week 24. Major secondary endpoints (week 24) were achievement of complete renal response (CRR), sustained reduction in steroid dose (≤10 mg/day prednisone/equivalent) from weeks 16–24, UPCR <0.5 mg/mg; <0.75 mg/mg, time to achieving CRR, and time to treatment failure. Safety was assessed through the end of the study.

**Results:**

Following enrolment challenges (COVID-19 pandemic; Ukraine/Russia crisis), the sponsor terminated the study early; 33 participants were randomized (placebo, *n* = 16; guselkumab, *n* = 17). At week 24, 56.3% (9/16) in the placebo group and 35.3% (6/17) in the guselkumab group achieved the primary endpoint. No apparent differences were observed in the secondary endpoints. Through end-of-study, 75% of placebo patients and 71% of guselkumab patients reported ≥1 adverse event; most were of mild-to-moderate severity.

**Conclusion:**

Guselkumab+background therapy did not demonstrate superior reduction in proteinuria *vs* placebo+background therapy in this small cohort of patients with active LN. Safety results were consistent with the known safety profile of guselkumab.

**Trial registration:**

ClinicalTrials.gov: NCT04376827.

Rheumatology key messages

- A phase 2 study evaluated the efficacy of guselkumab (IL-23p19-subunit inhibitor) in active LN.
- The study was terminated early (enrollment challenges), limiting the interpretation of the results.
- Guselkumab+background therapy was not superior in reducing proteinuria *vs* placebo+background therapy in this small cohort.

## Introduction

LN is the most common severe manifestation of SLE, affecting up to 60% of patients [1]. Kidney disease often develops early in SLE; in a retrospective analysis, nearly two-thirds of patients developed kidney disease within 5 years of SLE onset [2]. Kidney involvement, particularly kidney failure, is associated with reduced life expectancy in patients with SLE [3]. The clinical manifestations of LN are variable, ranging from asymptomatic proteinuria to nephritic and nephrotic syndromes and kidney failure [4, 5]. Histological evidence of LN is often present in patients with SLE, even in the absence of clinical symptoms of kidney disease [6]. International Society of Nephrology/Renal Pathology Society (ISN/RPS) Class III and Class IV LN are characterized by rapid progression to irreversible nephron loss and kidney failure if left untreated [1, 7].

The ultimate goal of LN therapy is preventing progression to chronic kidney disease, kidney failure and death [8]. Despite considerable advances in understanding the pathogenesis of LN and the introduction of new treatments [9, 10], kidney survival rates for patients with LN in developed countries plateaued in the mid-1990s and fell in the late 2000s [11]. Historically, kidney failure develops in 5–20% of patients with LN within 10 years of diagnosis [1], suggesting an unmet need in preventing progression.

Treatment for LN is largely based on histological class and activity [1, 8]. Patients with Class I and II may generally be monitored and do not need specific treatment unless presenting with nephrotic syndrome [8]. Current treatment guidelines for Class III and IV LN recommend combined immunosuppressives and glucocorticoids with or without belimumab (monoclonal antibody inhibiting B lymphocyte stimulator) or voclosporin (calcineurin inhibitor) [12], administered as induction therapy followed by long-term maintenance therapy, with the goals of reducing proteinuria, preserving kidney function and preventing flares, while reducing morbidity, mortality and medication-induced toxicity [1]. Response to therapy (stabilization or improvement in kidney function with ≥50% reduction in proteinuria within the first 6 months) has been identified as a strong prognostic indicator of long-term kidney survival, highlighting the benefit of tight disease control [13, 14]. The combination of CYC or MMF/mycophenolic acid (MPA), with a high-dose glucocorticoid was previously the standard-of-care for patients with active LN. Globally, belimumab [15] and voclosporin [16] are approved, in combination with standard-of-care immunosuppressive therapy, for adults with active LN; these treatments were approved by the US Food and Drug Administration in 2020 and 2021, respectively. However, in phase 3 studies of these treatments, substantial proportions of patients did not achieve complete renal response (CRR) [17, 18]. Thus, there remains a significant unmet need for therapies that can provide sustained remission in this patient population.

The pathogenesis of LN is multifactorial, with involvement of genetic, environmental and hormonal elements. Patients with SLE have elevated levels of IL-23 relative to healthy controls, and IL-23 levels have been strongly correlated with proteinuria [19–21]. Additionally, among patients with biopsy-confirmed active LN who received 6 months of immunosuppressive induction therapy, non-responders had significantly higher IL-23 serum levels compared with those achieving complete response [22]. In an analysis utilizing lupus-prone mice, exposure to an antibody inhibiting the IL-23p19 subunit resulted in less severe proteinuria relative to controls [23]. Taken together, these data support the scientific rationale for further evaluation of an IL-23p19-subunit inhibitor in patients with LN.

Guselkumab is a fully human mAb that selectively inhibits the IL-23p19 subunit and is approved globally for treating adults with moderate-to-severe plaque psoriasis and active PsA [24]. A phase 2, randomized, placebo-controlled study (A Multicentre, Randomized, Double-blind, Placebo-Controlled, Parallel-Group Study of Guselkumab in Subjects with Active Lupus Nephritis; ORCHID-LN) was initiated (November 2020) to evaluate the safety and efficacy of guselkumab in patients with active LN. However, owing to enrolment challenges, the study was terminated early by the sponsor. Herein, we report the efficacy and safety findings from the abbreviated ORCHID-LN study.

## Methods

### Participants

Eligible participants were adults (18–75 years inclusive) meeting the ACR/EULAR classification criteria for SLE with a kidney biopsy demonstrating active proliferative LN, defined as ISN/RPS Class III or IV [7, 25] (with or without Class V membranous nephritis) obtained within the 6 months prior to or during screening. A positive test result for ANA (titre ≥1:80) or anti-dsDNA antibodies (>75 IU/ml), both analysed by a central laboratory, was required. Participants must have received MMF/MPA for ≥8 weeks (current dose: MMF ≤3 g/day; MPA ≤2.2 g/day) at randomization and been receiving glucocorticoids for ≥6 weeks with a stable dose ≥2 weeks prior to randomization. Concomitant therapy with angiotensin-converting enzyme inhibitors (ACEI)/angiotensin II receptor blockers (ARBs) (stable dose ≥2 weeks prior to randomization) or antimalarials (treated for ≥12 weeks with stable dose ≥6 weeks at randomization) was permitted. A urine protein-to-creatinine ratio (UPCR) ≥1 mg/mg, assessed in two separate samples (first morning urine void specimens), was required following ≥8 weeks of MMF/MPA therapy and after stable glucocorticoid dosing was achieved. Additional details on exclusion criteria and permitted prior medications are reported in Supplementary Data S1, available at *Rheumatology* online. ORCHID-LN was conducted in accordance with principles of the Declaration of Helsinki. The study protocol was approved by the Institutional Review Board or Ethics Committee at each site. All participants gave written informed consent.

### Study design

**Figure 1.**
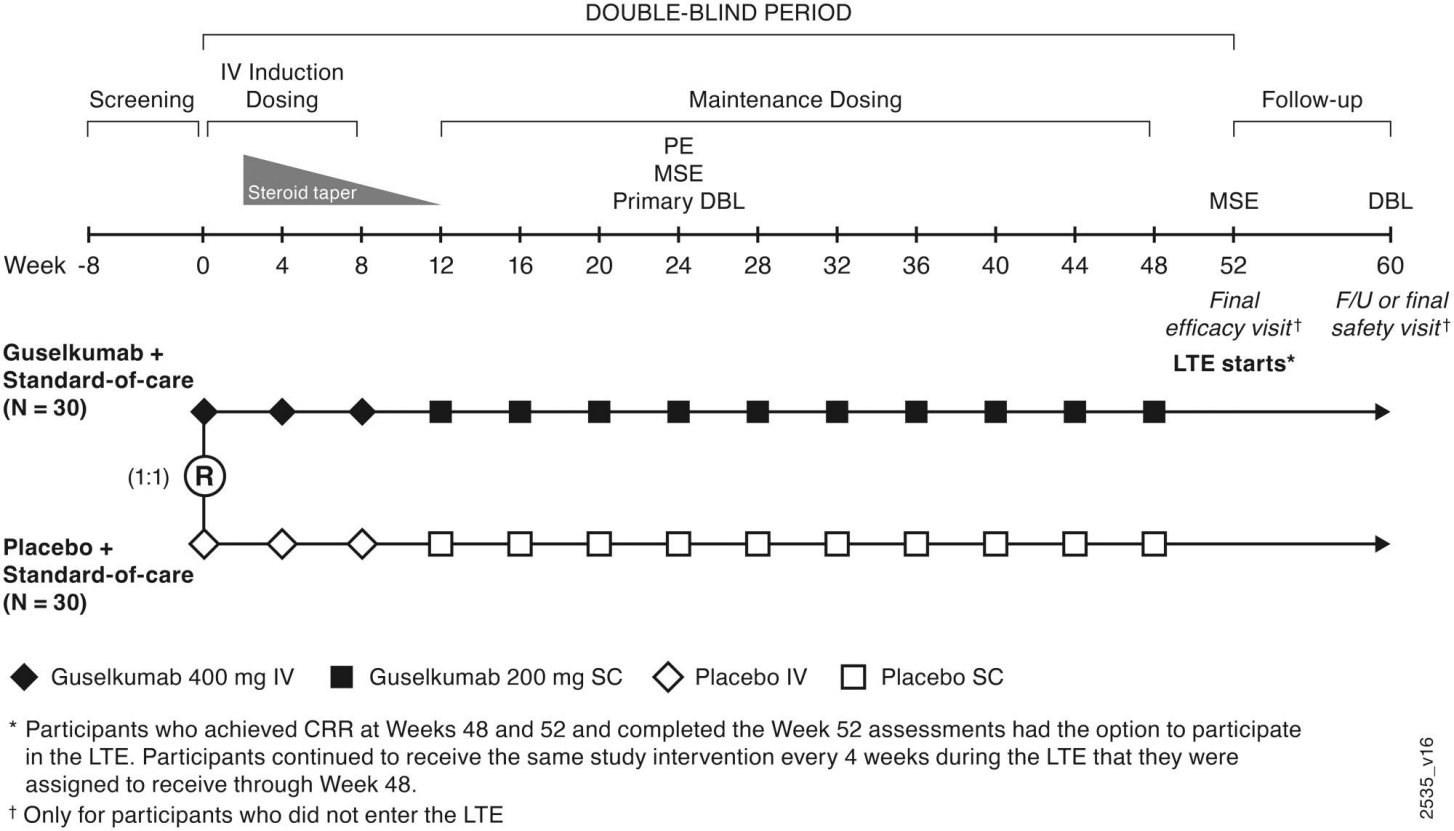
ORCHID-LN study schema. ORCHID-LN: A Multicentre, Randomized, Double-blind, Placebo-Controlled, Parallel-Group Study of Guselkumab in Subjects with Active Lupus Nephritis; CRR: complete renal response; DBL: database lock; F/U: follow-up; IV: intravenous; LTE: long-term extension; MSE: major secondary endpoint; PE: primary endpoint; R: randomization; SC: subcutaneous

The guselkumab dosing regimen selected for evaluation was informed by treatment guidelines for CYC in patients with active Class III and Class IV LN [8] and safety findings with guselkumab dosing regimens evaluated in patients with psoriasis, PsA, Crohn’s disease and ulcerative colitis [24, 26]. Eligible participants were randomly assigned (1:1) to receive i.v. infusions of guselkumab 400 mg at weeks 0, 4 and 8 followed by s.c. injections of guselkumab 200 mg every 4 weeks (Q4W) from week 12 through week 48 or placebo (i.v. at weeks 0, 4 and 8 and s.c. Q4W from week 12 through week 48) (Fig. 1). Participants achieving CRR at week 52 had the option to enter the long-term extension (LTE) and continue receiving their randomized study intervention through week 148.

At baseline, participants remained on their background treatment of MMF/MPA and glucocorticoids. All participants were required to adhere to a prespecified glucocorticoid dose-tapering schedule, as closely as possible, from week 2 through week 12, with a maximum final dosage of 5 mg (prednisone equivalent) orally once daily, and remain at this dosage through week 24. Concomitant use of ACEI/ARBs, antimalarials, NSAIDs or other analgesics, and select topical medications for cutaneous disease was permitted at stable doses.

Participants experiencing a flare (defined as an increase in proteinuria and/or serum creatinine concentration, abnormal urine sediment or a reduction in creatinine clearance rate as a result of active disease that requires a change in an immunomodulatory medication for LN beyond changes permitted per protocol) during the study were to be discontinued from study intervention.

### Assessments

LN disease activity through week 24 was evaluated by UPCR (spot urine; for all proteinuria analyses) and estimated glomerular filtration rate (eGFR). Urine samples were collected at baseline and at 4-weekly intervals through week 24. Baseline disease activity was assessed by the investigator using the SLE Disease Activity Index 2000 (SLEDAI-2K) [27, 28].

Serum samples were collected for blood chemistry assessments, including eGFR and measurement of serum guselkumab concentrations, and for evaluation of antibodies to guselkumab using validated assay methods. Serum guselkumab concentrations were determined using a validated, specific and sensitive electrochemiluminescence immunoassay (ECLIA) method [Meso Scale Discovery (MSD^®^) platform; Gaithersburg, MD, USA]. The presence of antibodies to guselkumab was assessed using a drug-sensitive ECLIA method (MSD platform).

Adverse events (AEs) were monitored throughout the study. AEs of interest included serious AEs (SAEs), infections, injection-site and infusion reactions, malignancies, and major adverse cardiovascular events (MACE; cardiovascular death, nonfatal myocardial infarction and nonfatal stroke).

### Statistical methods

The primary endpoint was the proportion of participants achieving ≥50% decrease in proteinuria from baseline at week 24. Sample size calculations accounted for a placebo response rate of 49%, based on results from a phase 2 study of voclosporin (ClinicalTrials.gov: NCT02141672). Together with an assumption of a 30% difference in response rates between the treatment groups, the planned sample size of 60 participants (30 in each treatment group) was estimated to provide ∼90% power (α = 0.10, one-sided) to detect a significant difference between treatment groups. However, following enrolment challenges from the COVID-19 pandemic and the Ukraine/Russia crisis, the sponsor terminated the study early, and the resulting population size of 33 participants lacked sufficient power to differentiate between the treatment groups. Although the final planned efficacy assessment in the main study period was at week 52, results through week 24 are reported herein owing to limited data availability.

Major secondary endpoints at week 24 included the proportions of participants achieving CRR (defined as meeting all of the following criteria: UPCR <0.5 mg/mg; eGFR ≥60 ml/min/1.73 m^2^ or no decrease ≥20% from baseline; and prednisone dosage ≤10 mg/day); a sustained reduction in glucocorticoid dose ≤10 mg/day of prednisone or equivalent from week 16 to week 24; UPCR <0.5 mg/mg; and UPCR <0.75 mg/mg; and the time to achievement of CRR. Time to treatment failure was also a major secondary endpoint and defined as an increase in concomitant glucocorticoid dosage (either exceeding the baseline level at any time or >10 mg/day prednisone equivalent after week 12); initiation or increase in dosage of other LN medications or immunosuppressive agents; or initiation of any prohibited medications. Exploratory *post hoc* analyses assessed mean absolute and percent changes in UPCR through week 24 by treatment group.

At the time of study termination, all enrolled participants had either received their week 20 study drug administration and completed the week 24 efficacy and safety assessments or discontinued study treatment. Analyses of efficacy endpoints through week 24 included all randomized participants who received ≥1 administration of study drug. For the primary and major secondary endpoints (with the exception of time to treatment failure), participants were considered to be nonresponders if they discontinued study treatment for any reason (excluding COVID-19) or met the treatment failure criteria described above with the distinction that initiation/increase in other LN medications or immunosuppressive agents was only prohibited within 8 weeks prior to the endpoint.

A generalized linear model with logit link for a binary mixed model for repeated measures (MMRM) was used to analyse the primary and all dichotomous major secondary endpoints. The model included all available observed data after applying intercurrent events, adjusting for treatment, visit, region, baseline UPCR level (<3 mg/mg and ≥3 mg/mg) and treatment by visit interaction. The secondary endpoints of time to achievement of CRR and time to treatment failure were assessed using Kaplan–Meier curves to calculate the hazard ratio (HR) and corresponding 80% CI using the Cox proportional hazards model adjusting for region and baseline UPCR (<3 mg/mg or ≥3 mg/mg). No adjustments were made for multiple comparisons, and all *P*-values presented herein are nominal.

Safety analyses included all randomized participants who received ≥1 administration of study drug. All AEs occurring from baseline through study termination were summarized by actual treatment received. Pharmacokinetic and immunogenicity analyses included data from all participants who received ≥1 administration of study drug and had ≥1 valid post-baseline serum sample.

## Results

### Patient disposition and baseline characteristics

ORCHID-LN was conducted from 18 November 2020 through 1 February 2023 in 20 centres across 9 countries (Table 1). At the time of study termination, 33 participants had been enrolled and randomized to receive placebo plus background therapy (*n* = 16) or guselkumab plus background therapy (*n* = 17). Baseline demographic and disease characteristics were generally similar between the treatment groups with some exceptions (Table 1). In both treatment groups, the median and mean duration of SLE were ∼7 and 8 years, respectively. The median duration of LN was 3.8 years in the placebo group and 6.3 years in the in the guselkumab group; the respective mean LN durations were 6.8 and 6.4 years. Most participants were classified (by kidney biopsy) as having either Class III (placebo 37.5%; guselkumab 23.5%) or Class IV (placebo 50.0%; guselkumab 58.8%) LN. One (6.3%) participant in the placebo group and three (17.6%) in the guselkumab group had both Class III and V LN; one (6.3%) participant in the placebo group and none in the guselkumab group had both Class IV and V LN.

**Table 1.** Baseline demographic and disease characteristics of ORCHID-LN participants^a^

	Placebo	Guselkumab	Total
Participants, *N*	16	17	33
Sex, *n* (%)			
Female	14 (87.5)	15 (88.2)	29 (87.9)
Male	2 (12.5)	2 (11.8)	4 (12.1)
Age, years	38.8 (11.7)	35.3 (10.1)	37.0 (10.9)
Race, *n* (%)			
American Indian or Alaska Native	1 (6.3)	2 (11.8)	3 (9.1)
Asian	3 (18.8)	1 (5.9)	4 (12.1)
White	12 (75.0)	14 (82.4)	26 (78.8)
Hispanic or Latino, *n* (%)	4 (25.0)	7 (41.2)	11 (33.3)
SLE disease duration, years			
Mean (s.d.)	8.0 (6.3)	7.8 (5.5)	7.9 (5.8)
Median (IQR)	6.8 (2.1; 11.5)	7.0 (3.7; 11.0)	7.0 (2.5; 11.2)
LN disease duration, years			
Mean (s.d.)	6.8 (6.6)	6.4 (5.1)	6.6 (5.8)
Median (IQR)	3.8 (1.3; 11.3)	6.3 (1.6; 10.0)	4.6 (1.6; 11.0)
Flare history, *n* (%)			
Yes	13 (81.3)	13 (76.5)	26 (78.8)
No	3 (18.8)	4 (23.5)	7 (21.2)
SLEDAI-2K score, excluding renal items (0–89)^b^	6.6 (3.4)	7.4 (4.6)	7.0 (4.0)
Kidney biopsy ISN/RPS class, *n* (%)			
Class III	6 (37.5)	4 (23.5)	10 (30.3)
Class III + V	1 (6.3)	3 (17.6)	4 (12.1)
Class IV	8 (50.0)	10 (58.8)	18 (54.5)
Class IV + V	1 (6.3)	0	1 (3.0)
UPCR, mg/mg			
Mean (s.d.)	2.7 (1.8)	2.8 (2.3)	2.7 (2.1)
Median (IQR)	2.1 (1.4; 4.1)	1.8 (1.5; 3.9)	1.9 (1.4; 4.0)
eGFR, ml/min/1.73 m^2^			
Mean (s.d.)	80.0 (29.8)	88.1 (34.4)	84.2 (32.0)
Median (IQR)	79.9 (61.4; 97.6)	91.5 (63.9; 105.2)	83.8 (33.3; 174.2)
<30, *n* (%)	0	0	0
≥30 and <60, *n* (%)	4 (25.0)	4 (23.5)	8 (24.2)
≥60 and <90, *n* (%)	7 (43.8)	4 (23.5)	11 (33.3)
≥90, *n* (%)	5 (31.3)	9 (52.9)	14 (42.4)
ANA, (titre ≥1:80), *n* (%)	16 (100)	17 (100)	33 (100)
Anti-dsDNA, *n* (%)			
Borderline (30–75 IU/ml)	2 (12.5)	3 (17.6)	5 (15.2)
Positive (>75 IU/ml)	7 (43.8)	8 (47.1)	15 (45.5)
Concomitant medications			
Antimalarials^c^, *n* (%)	11 (68.8)	9 (52.9)	20 (60.6)
Dose, mg/day	327.3 (100.9)	282.5 (112.9)	307.1 (106.0)
MMF or mycophenolic acid^d^, *n* (%)	16 (100)	17 (100)	33 (100)
Dose, g/day	2.2 (0.5)	2.1 (0.6)	2.2 (0.6)
Oral glucocorticoids^e^, *n* (%)	16 (100)	17 (100)	33 (100)
Dose, mg/day	22.8 (15.4)	22.1 (17.6)	22.4 (16.3)
ACEI or ARB, *n* (%)	13 (81.3)	7 (41.2)	20 (60.6)
NSAIDs, *n* (%)	3 (18.8)	2 (11.8)	5 (15.2)
Other SLE/LN medications, *n* (%)	4 (25.0)	4 (23.5)	8 (24.2)
Prior medications for lupus, *n* (%)			
Oral glucocorticoids	16 (100)	17 (100)	33 (100)
MMF or mycophenolic acid	15 (93.8)	16 (94.1)	31 (93.9)
Antimalarials	15 (93.8)	13 (76.5)	28 (84.8)
Systemic CYC	8 (50.0)	9 (52.9)	17 (51.1)
AZA or 6-MP	3 (18.8)	6 (35.3)	9 (27.3)
MTX	2 (12.5)	4 (23.5)	6 (18.2)
Voclosporin	0	1 (5.9)	1 (3.0)
Tacrolimus	0	1 (5.9)	1 (3.0)
LEF	0	1 (5.9)	1 (3.0)
Rituximab	1 (6.3)	0	1 (3.0)
Belimumab	0	0	0
Mizoribine	0	0	0
Other biologic agents	1 (6.3)	2 (11.8)	3 (9.1)
Other SLE medications	11 (68.8)	9 (52.9)	20 (60.6)

Data reported as mean (s.d.), unless otherwise noted.

a ORCHID-LN was conducted in 20 centres across 9 countries and enrolled 33 participants as follows: Argentina *n* = 9, Mexico *n* = 4, Poland *n* = 0, Russian Federation *n* = 3, Spain *n* = 0, Taiwan *n* = 3, Thailand *n* = 1, Ukraine *n* = 12, USA *n* = 1.

b *N* = 15 in the placebo group.

c Includes chloroquine, HCQ and quinacrine; dose of antimalarials converted to HCQ equivalence.

d Dose of mycophenolic acid converted to MMF equivalence.

e Dose of oral glucocorticoids converted to prednisone equivalence. ACEI: angiotensin-converting enzyme inhibitor; ARB: angiotensin receptor blocker; eGFR: estimated glomerular filtration rate; ISN/RPS: International Society of Nephrology/Renal Pathology Society; IQR: interquartile range; ORCHID-LN: A Multicentre, Randomized, Double-blind, Placebo-Controlled, Parallel-Group Study of Guselkumab in Subjects with Active Lupus Nephritis; SLEDAI-2K: SLEDAI 2000; UPCR: urine protein-creatine ratio; 6-MP: 6-mercaptopurine.

The median (interquartile range) UPCR was 2.1 mg/mg (1.4, 4.1) among participants in the placebo group and 1.8 mg/mg (1.5, 3.9) in the guselkumab group (Table 1). The median (interquartile range) eGFR (ml/min/1.73 m^2^) was 79.9 (61.4, 97.6) in the placebo group and 91.5 (63.9, 105.2) in the guselkumab group. All participants in the placebo and guselkumab groups received prior oral glucocorticoids for lupus; 93.8% and 94.1%, respectively, received prior MMF/MPA therapy, and 93.8% and 76.5%, respectively, received prior antimalarials (Table 1). Nine participants (placebo *n* = 3; guselkumab *n* = 6) reported prior use of AZA/6-MP, one (guselkumab) received tacrolimus and one (placebo) previously received rituximab; no participant reported prior therapy with belimumab or mizoribine. At baseline, all participants were receiving MMF and oral glucocorticoids; mean daily doses were similar between treatment groups. Concomitant use of antimalarials was more common in the placebo than in the guselkumab group (68.8% *vs* 52.9%), and 81.3% in the placebo group were receiving an ACEI or ARB compared with 41.2% in the guselkumab group. Concomitant NSAID use was reported by 18.8% of participants in the placebo group and 11.8% in the guselkumab group.

**Figure 2.**
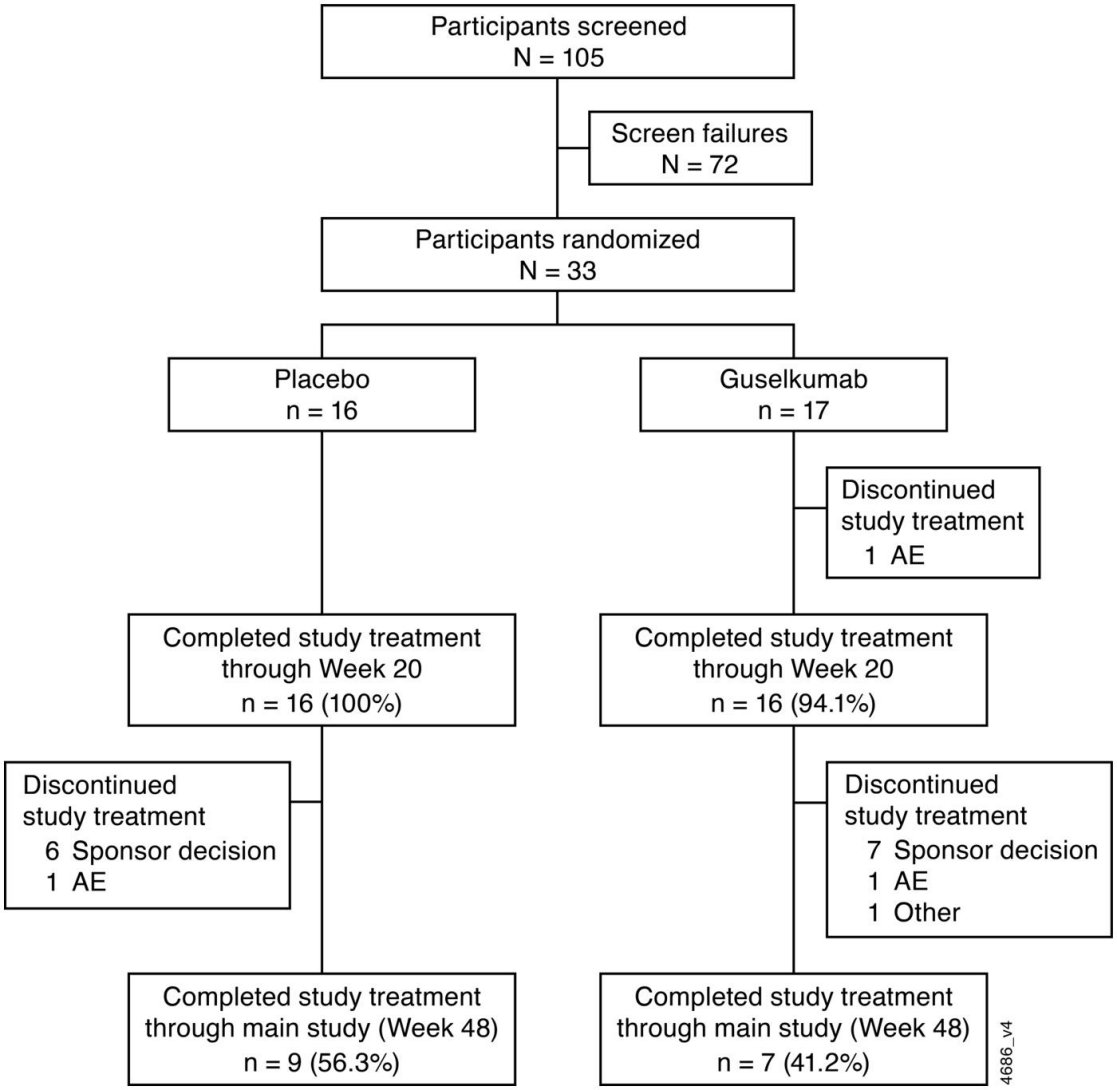
Participant disposition through week 48 in ORCHID-LN. ORCHID-LN: A Multicentre, Randomized, Double-blind, Placebo-Controlled, Parallel-Group Study of Guselkumab in Subjects with Active Lupus Nephritis; AE: adverse event

Through week 24, no participant in the placebo group and one (3.0%) in the guselkumab group discontinued study treatment (due to an AE of SLE flare). Between weeks 24 and 48, 7 (43.8%) participants in the placebo group and 9 (58.8%) in the guselkumab group discontinued study treatment (Fig. 2); of these, 13 discontinued (39.4%) due to the study termination by sponsor, and 3 (9.1%) discontinued as a result of an AE. Of the remaining 16 participants who completed study treatment through the main study (week 48), 5 (placebo, *n* = 4; guselkumab, *n* = 1) entered the LTE; none completed treatment in the LTE prior to study termination.

### Efficacy

**Figure 3.**
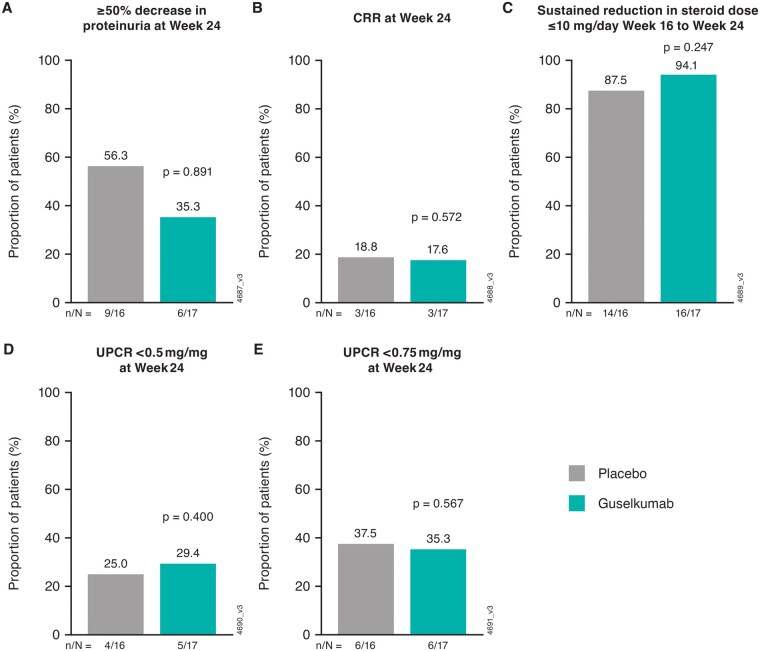
Proportions of ORCHID-LN participants achieving key efficacy endpoints at week 24. (**A**) ≥50% decrease in proteinuria (primary endpoint), (**B**) CRR, (**C**) sustained reduction in glucocorticoid dose ≤10 mg/day from week 16, (**D**) UPCR <0.5 mg/mg, and (**E**) UPCR <0.75 mg/mg. ORCHID-LN: A Multicentre, Randomized, Double-blind, Placebo-Controlled, Parallel-Group Study of Guselkumab in Subjects with Active Lupus Nephritis;CRR: complete renal response; UPCR: urine protein-to-creatinine ratio

**Figure 4.**
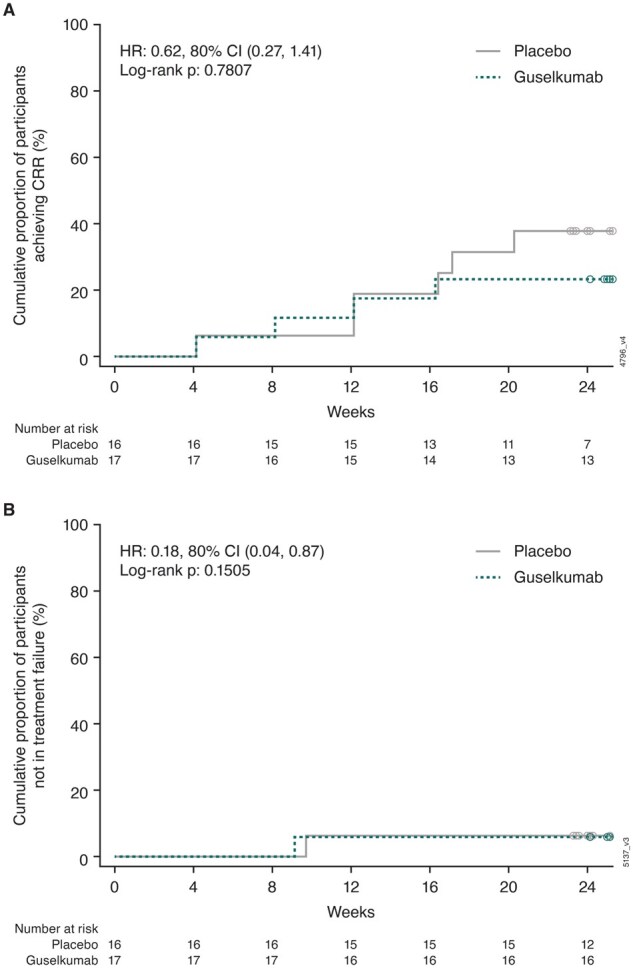
Time to achievement of CRR (**A**) and time to treatment failure^a^ (**B**) through week 24. ^a^Treatment failure defined as: an increase in concomitant glucocorticoid dosage (either exceeding the baseline level at any time or >10 mg/day prednisone equivalent after week 12); initiation or increase in dosage of other LN medications or immunosuppressive agents; or initiation of any prohibited medications. CRR: complete renal response; HR: hazard ratio

At week 24, 56.3% (9/16) of participants receiving placebo plus background therapy and 35.3% (6/17) (*P* = 0.891) receiving guselkumab plus background therapy achieved the primary endpoint of ≥50% decrease in proteinuria from baseline (Fig. 3). All but two participants, one in each treatment group, were able to achieve glucocorticoid tapering to 5 mg/day by week 24. There were no notable differences between the placebo and guselkumab groups, respectively, in response rates for achieving CRR at week 24 [3/16 (18.8%) and 3/17 (17.6%)], sustained reduction in glucocorticoid dose of ≤10 mg/day of prednisone or equivalent from week 16 to week 24 [14/16 (87.5%) and 16/17 (94.1%)], UPCR <0.5 mg/mg at week 24 [4/16 (25.0%) and 5/17 (29.4%)], and UPCR <0.75 mg/mg at week 24 [6/16 (37.5%) and 6/17 (35.3%)]. The HR (80% CI) for achieving CRR through week 24 with guselkumab *vs* placebo was 0.62 (0.27, 1.41) (*P* = 0.7807) (Fig. 4), and the HR (80% CI) for time to treatment failure through week 24 was 0.18 (0.04, 0.87) (Fig. 4).

In exploratory *post hoc* analyses, there were no apparent differences between guselkumab and placebo when assessing the absolute mean change and mean percent change in UPCR from baseline through week 24 (Supplementary Figs S1 and S2, available at *Rheumatology* online).

### Safety

All 33 randomized participants received ≥1 study drug administration and were included in the safety analyses. The mean duration of safety follow-up was 56.7 weeks in the placebo group and 52.5 weeks in the guselkumab group (Table 2); through the final safety-follow up, 75% and 71%, respectively, reported ≥1 AE. One SAE occurred in each treatment group (placebo: basal ganglia stroke; guselkumab: worsening of LN). Infections were the most common AE, with five (31.3%) participants in the placebo group and six (35.3%) in the guselkumab group reporting ≥1 infection, including herpes zoster (placebo *n* = 2; guselkumab *n* = 1) and urinary tract infection (placebo *n* = 2; guselkumab *n* = 2). Three cases of COVID-19 occurred, all in the placebo group. One participant experienced a MACE (placebo: basal ganglia stroke). There were no serious infections, opportunistic infections, hypersensitivity reactions, cases of active TB, venous thromboembolic events, malignancies or deaths in either treatment group.

**Table 2.** Adverse events through study termination in ORCHID-LN

	Placebo	Guselkumab
Participants, *N*	16	17
Mean duration of follow-up, weeks	56.7	52.5
Participants with ≥1, *n* (%):		
AE	12 (75.0)	12 (70.6)
SAE	1 (6.3)	1 (5.9)
Infection^a^	5 (31.3)	6 (35.3)
Bacterial pneumonia	1 (6.3)	0
Bacterial vaginosis	0	1 (5.9)
Bronchitis	0	1 (5.9)
Chronic pyelonephritis	0	1 (5.9)
COVID-19	3 (18.8)	0
Herpes zoster	2 (12.5)	1 (5.9)
Influenza	0	2 (11.8)
Upper respiratory tract infection	1 (6.3)	0
Urinary tract infection	2 (12.5)	2 (11.8)
Serious infection	0	0
COVID-19-related AEs	3 (18.8)	0
Infusion-site reactions^b^	1 (6.3)	0
Injection-site reactions	1 (6.3)	0
Malignancies	0	0
AEs leading to discontinuation of study agent	1 (6.3)	2 (11.8)
Deaths	0	0

Data reported as *n* (%), *n*/*N* (%) or mean, unless otherwise noted.

a Assessed by the investigator.

b Temporally associated with infusion (during or within 1 h after infusion). AE: adverse event; ORCHID-LN: A Multicentre, Randomized, Double-blind, Placebo-Controlled, Parallel-Group Study of Guselkumab in Subjects with Active Lupus Nephritis; SAE: serious adverse event.

### Pharmacokinetics

Sixteen participants received ≥1 guselkumab administration and had ≥1 evaluable post-baseline serum sample. One participant whose serum guselkumab levels were below the lower limit of quantification (i.e., 0.01 μg/ml) was excluded; an investigation was performed, and there was no indication of study agent administration errors or quality issues with the study interventions. Following i.v. administration of guselkumab 400 mg at week 0, the median serum guselkumab concentration 1 h post-infusion was 141.9 μg/ml. At week 24 (primary endpoint) following s.c. maintenance dosing of guselkumab 200 mg Q4W starting at week 12, the median trough serum guselkumab concentration was 5.4 μg/ml.

### Immunogenicity

All 17 participants randomized to guselkumab received ≥1 guselkumab administration and had evaluable post-baseline serum samples. Through week 24, one participant (5.9%) tested positive for antibodies to guselkumab; this participant tested negative for neutralizing antibodies. No additional participants tested positive for antibodies to guselkumab from weeks 24–60.

## Discussion

Following early termination of this phase 2 study with ∼50% of the study enrolled, the resulting sample size was insufficiently powered to detect statistically significant differences between the treatment groups. Available results did not demonstrate superiority of guselkumab *vs* placebo in participants with active LN receiving background therapy across all endpoints assessed through week 24. In addition to the inadequate sample size, interpretation of these results may be further confounded by imbalances in baseline characteristics between the treatment groups, including concomitant medications and the duration of LN. Specifically, 81% of participants in the placebo group were receiving concomitant ACEI *vs* 41% in the guselkumab group. ACEI have been shown to have a renoprotective effect in patients with SLE [29], and current treatment guidelines support the use of renin–angiotensin–aldosterone blockade (ACEI/ARB) in these patients with LN [13].

Both ACEI and ARB have been shown to reduce proteinuria independent of their effects on hypertension [30]. ACEI therapies block the breakdown of bradykinin (an efferent arteriolar vasodilator), leading to decreases in both systemic and glomerular capillary pressure [31]. Decreases in intraglomerular pressure and glomerular hyperfiltration then lead to a reduction in proteinuria [32]. Significant improvements in proteinuria and serum albumin levels have been observed after 6 and 24 months of treatment with ACEI/ARB in patients with persistent proteinuria (UPCR >1 g/day) despite resolution of acute LN following immunosuppressive treatment [33]. Furthermore, meta-analyses have noted that treatment with ACEI, ARB or both have beneficial effects on reducing proteinuria in patients with renal disease [34, 35]. Mean baseline UPCR levels in the current study were lower than those observed in other recent trials of participants with LN [17, 18], and overall, levels were considered generally mild (median ∼2 mg/mg). The disparate use of ACEI in the treatment groups, relatively mild proteinuria in ORCHID-LN participants and differences in the duration of LN between the treatment groups may have limited the ability to accurately evaluate the effects of guselkumab in this population.

The types and frequencies of AEs were consistent with the known safety profile of guselkumab in patients with psoriasis and PsA [36–39]. Although limited by the small sample size in ORCHID-LN, no new or unexpected safety events were observed in this study despite participants more commonly receiving glucocorticoids at higher mean doses in comparison with the psoriasis and PsA study populations [36–39]. There were no serious infections, opportunistic infections, hypersensitivity reactions, cases of active TB, venous thromboembolic events, malignancies or deaths in either treatment group.

The ORCHID-LN findings were primarily limited by effects from early study termination, i.e., small sample sizes and efficacy assessments restricted to a relatively short time period of 6 months. Additionally, the study population was predominantly White and female, thereby limiting assessments in other patient subgroups [40–45]. The frequency of prior (85%) and concomitant use (61%) of antimalarials by ORCHID participants was slightly lower than, but generally consistent with, those in an analysis of electronic health records in the USA in which 86% of patients with LN had a history of and 63% were currently receiving antimalarials [46]. Additionally, patients who were being followed by a rheumatologist were significantly more likely to be prescribed an antimalarial than those followed by a nephrologist. Given that most of the ORCHID-LN study investigators were nephrologists, this may have affected the frequency of antimalarial use in the ORCHID-LN population.

Dysregulation of the IL-23/Th17 pathway has been implicated in several autoimmune diseases, including SLE/LN, and inhibition of IL-23 and IL-17A with guselkumab and secukinumab, respectively, has demonstrated efficacy in improving the signs and symptoms of psoriasis [47] and PsA [48]. In 2019, just prior to the ORCHID-LN study, a separate phase 3, randomized, double-blind, placebo-controlled study (SELUNE) was initiated to evaluate the efficacy and safety of secukinumab in patients with active LN, but was terminated early by the study sponsor (Novartis) following a futility analysis (ClinicalTrials.gov: NCT04181762). Together with the limited findings from ORCHID-LN, this would suggest that although expression of IL-23 and IL-17 may be aberrant in patients with LN, the role of the IL-23/Th17 pathway in LN is unclear. Thus, identification of effective therapies to reduce the burden of LN remains an unmet need.

## Supplementary Material

keae647_Supplementary_Data

## Data Availability

Data are available on reasonable request. The data sharing policy of Janssen Pharmaceutical Companies of Johnson & Johnson is available at https://www.janssen.com/clinical-trials/transparency. As noted on this site, requests for access to the study data can be submitted through Yale Open Data Access (YODA) Project site at http://yoda.yale.edu.
