# Supplementary material for: Efficacy and safety of guselkumab in patients with active lupus nephritis: results from a phase 2, randomized, placebo-controlled study
Source: Rheumatology (Oxford). 2024 Dec 2;64(5):2731–40. doi: 10.1093/rheumatology/keae647 (PMC12048075; doi:10.1093/rheumatology/keae647)
Supplement: keae647_Supplementary_Data [file keae647_supplementary_data.docx]

**Supplementary Data S1**

ORCHID-LN Exclusion Criteria and Prior Medications Permitted.

Participants were excluded if they had a history of or current severe, progressive or uncontrolled kidney disease (other than active LN); isolated or predominant Class V LN (i.e., without coexistent Class III or IV nephritis); severe, progressive, or uncontrolled hepatic, hematological, gastrointestinal, pulmonary, cardiac, or neurological disease (either related or unrelated to SLE); required hemo- or peritoneal dialysis or were anticipated to require dialysis within 6 months; transplanted organ (other than corneal transplant performed >12 weeks before screening); or unstable or progressive manifestation of SLE that is likely to warrant escalation in therapy beyond permitted background medications. Eligible participants also could not have had other comorbidities that required ≥3 courses of systemic glucocorticoids within the previous 12 months; other inflammatory diseases that might confound the evaluations of efficacy; a history or suspected occurrence of drug-induced lupus or catastrophic antiphospholipid syndrome; or inherited complement deficiency or combined variable immunodeficiency.

Participants who had received prior therapy with one B-cell targeting agent (within 3 months prior to randomization), or more than one B-cell targeting agent, including belimumab (within 6 months prior to randomization), or any prior therapy with a B-cell depleting agent (eg, rituximab or obinutuzumab; within 12 months prior to randomization) or had evidence of continued B-cell depletion were not eligible. Additionally, participants could not have received prior therapy with IL-12 or IL-12/23 inhibitors (at any time); tumor necrosis factor inhibitors or agents targeting IL-1, IL-2, IL-6, IL-17, or cytotoxic T-lymphocyte associated protein 4 (within the greater of either 90 days or 5 half-lives); or systemic immunomodulatory agents other than the permitted prior or concomitant medications described above (e.g., leflunomide, methotrexate, tacrolimus, sirolimus, mizoribine, cyclosporine, and voclosporin [within 3 months prior to randomization], azathioprine/6-mercaptopurine [within 2 months prior to randomization]), or oral or IV cyclophosphamide (within 3 months prior to randomization).

**Supplementary Figure S1.** Change from baseline in UPCR through Week 24 in the guselkumab and placebo groups in the ORCHID-LN study. Box = 25^th^ and 75^th^ percentiles; whiskers = 5^th^ and 95^th^ percentiles; horizontal line = median; + = mean.

UPCR, urine protein-to-creatinine ratio

**
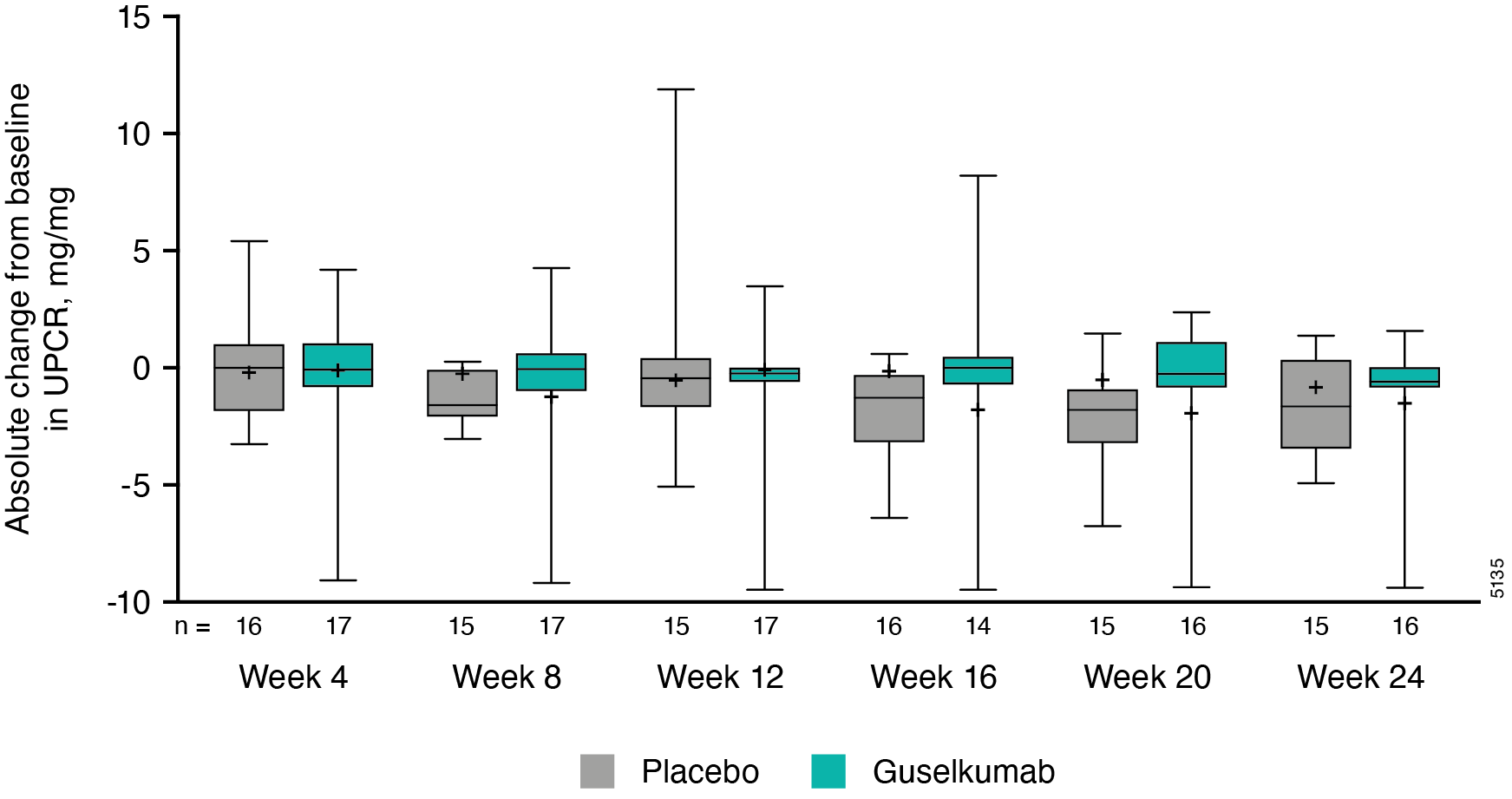
**

**Supplementary Figure 2.** Percent change from baseline in UPCR through Week 24 in the guselkumab and placebo groups in the ORCHID-LN study. Box = 25^th^ and 75^th^ percentiles; whiskers = 5^th^ and 95^th^ percentiles; horizontal line = median; + = mean.

UPCR, urine protein-to-creatinine ratio

**
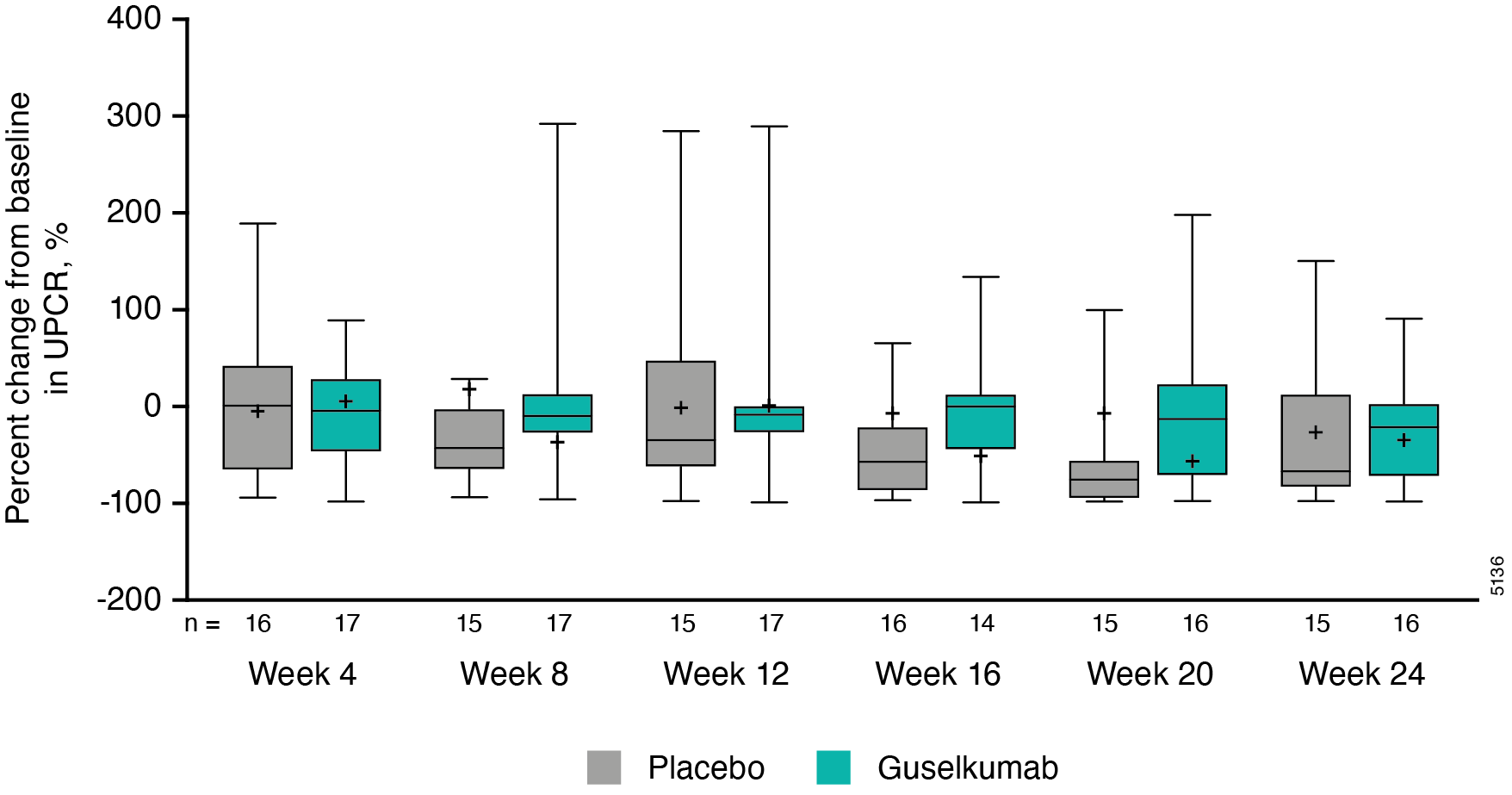
**
